# Mediating Role of Resilience in the Relationships Between Fear of Happiness and Affect Balance, Satisfaction With Life, and Flourishing

**DOI:** 10.5964/ejop.v15i2.1640

**Published:** 2019-06-07

**Authors:** Murat Yildirim

**Affiliations:** a Department of Neuroscience, Psychology and Behaviour, University of Leicester, Leicester, United Kingdom; Webster University Geneva, Geneva, Switzerland; University of Wroclaw, Wroclaw, Poland

**Keywords:** resilience, fear of happiness, affect balance, positive affect, negative affect satisfaction with life, flourishing

## Abstract

The present study was a first attempt to examine the mediating role of resilience in the relationships between fear of happiness and affect balance, satisfaction with life, and flourishing. Participants consisted of 256 Turkish adults (174 males and 82 females) and aged between 18 and 62 years (M = 36.97, SD = 9.02). Participants completed measures assessing fear of happiness, affect balance, satisfaction with life, and flourishing. The results showed that fear of happiness was negatively correlated with resilience, affect balance, satisfaction with life, and flourishing, while resilience was positively correlated with affect balance, satisfaction with life, and flourishing. The results of mediation analysis showed that (a) resilience fully mediated the effect of fear of happiness upon flourishing, and satisfaction with life, (b) partially mediated the effect of fear of happiness upon affect balance. These findings suggest that resilience helps to explain the associations between fear of happiness and affect balance, satisfaction with life, and flourishing. This study elucidates the potential mechanism behind the association between fear of happiness and indicators of well-being.

With the advent of positive psychology, researchers have focused not only on treating negative states (e.g. depression, anxiety) but also promoting positive states (e.g. happiness, life satisfaction), individual strengths and their relations to mental health, well-being and social prosperity (Seligman & Csikszentmihalyi, 2000). Positive psychology emphasises the importance of identifying and understanding factors associated with subjective well-being, psychological well-being, and flourishing.

Recent research identified new sets of variables that affect both subjective and psychological well-being. For example, fear of happiness is one of the variables that was found to be negatively associated with positive psychological variables. Fear of happiness refers to the notion that being happy results in bad things to happen and should be bewared of (Joshanloo, 2013). According to Joshanloo (2013), individuals who have high levels of fear of happiness may suppress their authentic positive emotions toward pursuing happiness to shun negative effects of positive states of mind (e.g. happiness, joy). Fear of happiness can be considered as a dysfunctional belief that may generate a tendency toward dampening of positive emotions. There is a variety of factors affecting people’s attitude toward experience of positive emotions. Joshanloo (2013) grounded the theoretical position of fear of happiness on a wide range of contexts including culture, religion, and superstitious beliefs. Giving specific focus to the cultural differences, Joshanloo et al. (2014) argued that fear of happiness is a reflection of culture and should be considered as a beliefs system in a culture. Therefore, culture is an important factor that affect people’s attitude toward perception and experience of positive emotions. Reasons for endorsing fear of happiness vary from person to person and culture to culture. However, four reasons appear to be universal for holding fear of happiness beliefs: (a) being happy stimulates unpleasant things to happen, (b) being happy morally makes you a worse person, (c) expressing positive emotions (e.g. happiness, cheerfulness) is bad for you and others, (d) seeking for happiness is bad for you and others (Joshanloo & Weijers, 2014).

Previous research provided evidence on the impact of fear of happiness on one’s well-being by showing that fear of happiness is negatively associated with subjective happiness, satisfaction with life, positive affect, autonomy, positive relations with others, self-acceptance, environmental mastery, personal growth, and purpose in life and that positively associated with negative affect (Joshanloo, 2013; Yildirim & Aziz, 2017; Yildirim & Belen, 2018). That is, individuals with higher levels of fear of happiness report lower levels of subjective and psychological well-being. Studies also demonstrated that higher scores on fear of happiness were related with higher scores on externality of happiness and lower scores on self-esteem (Yildirim, Barmanpek, & Farag, 2018). Furthermore, fear of happiness was found to be uniquely important to both subjective and psychological well-being after controlling for personality. Fear of happiness is directly responsible for regulating individual difference in the experience of lower positive affect and higher negative affect — components of subjective well-being — and lower positive relations with others, self-acceptance and autonomy — components of psychological well-being (Yildirim & Belen, 2018).

Well-being is a multifaceted concept of optimal experience, and functioning and generally derived from two broad approaches (Ryan & Deci, 2001). The first approach is *subjective well-being or happiness*, grounded on hedonism and that refers to the experience of pleasure and avoidance of pain (Kahneman, Diener, & Schwarz, 1999). The second approach is *psychological well-being or flourishing*, grounded on eudaimonia, and that refers to how to achieve the realization of one’s authentic potential, goals, meaning, and fulfilment in life (Ryff, 1989; Ryff & Keyes, 1995). Subjective well-being refers to cognitive and affective assessment of life (Diener, 1984, 2000). Put it differently, subjective well-being is defined as what individuals think and how they feel about their lives when they make cognitive and affective judgements about their existence (Seligman & Csikszentmihalyi, 2000). Subjective well-being is conceptualized as being a tripartite model that constitutes three different, yet associated elements: the presence of frequent positive emotions, the infrequent of negative emotions, and the satisfaction with life (Diener, Lucas, & Oishi, 2002; Diener, Scollon, & Lucas, 2003; Ryan & Deci, 2001). Cross-sectional, longitudinal, and experimental studies revealed that individual with high levels of subjective well-being are more successful across different life domains including but not limited to social relationship, marriage, income, work performance, and physical and mental health. Evidence also showed that happiness-related characteristics, resources, and successes are arisen as results of experiencing frequent positive affect (Lyubomirsky, King, & Diener, 2005). Furthermore, studies indicated that higher satisfaction with life is positively associated with adaptive psychological constructs (e.g. gratitude) and negatively associated with maladaptive psychological constructs (e.g. perceived stress) (Yildirim & Alanazi, 2018). Psychological well-being has been characterised as an array of psychological characteristics concerned with positive human functioning such as striving for excellence and engagement with life difficulties (Keyes, Shmotkin, & Ryff, 2002). Ryff (1989) proposed that psychological well-being can be defined as having six elements including, autonomy, positive relations with others, self-acceptance, environmental mastery, purpose in life and personal growth. These elements are theoretically and empirically independent yet related with one another and that an optimal achievement of psychological well-being requires striving each of the elements independently. Cross-sectional and longitudinal evidence suggested that psychological well-being is related with flexible and creative thinking, pro-social behaviour, physical health (Huppert, 2009), and meatal health (Ryff, Radler, & Friedman, 2015).

The concept of flourishing is presented as an inclusive and integrative theoretical framework to understand well-being by encompassing psychological and social well-being and refers to optimal level of well-being (Diener et al., 2010; Keyes, 2002). Flourishing as a psychosocial model of well-being is described as a composition of feeling good and functioning effectively and that includes multiple concepts of high levels of psychological well-being, emotional well-being, and social well-being (Huppert & So, 2013; Keyes, 2002). It also refers to a fundamental ingredient of well-being that leads to a positive human development (Huppert & So, 2013), and fulfilment and purpose in life (Seligman, 2011). Flourishing is not only the absence of mental health problems such as anxiety and depression, but also the opposite of mental health problem (Huppert & So, 2013). It can be conceived as a source of resilience, functioning as a protective factor against stressful life events (Keyes, 2002).

According to Huppert and So (2013), flourishing should be investigated in its own right to determine the correlates and causes of flourishing in order to understand positive aspects of human functioning. Seligman (2011) provided a theoretical foundation of flourishing by combining hedonic and eudaimonic approaches of well-being and grounded flourishing on five independent but related elements, known with the acronym of PERMA: Positive emotions (P), engagement (E), relationships (R), meaning (M), and achievement (A).

It is important to enhance flourishing in order to promote well-being. Evidence suggested that flourishing is related with psychosocial functioning including better emotional health, higher engagement with daily life activities, fewer work loss days, and days of work cutbacks (Keyes, 2002, 2007). Studies also showed that flourishing is positively associated with purpose in life, resilience, and high intimacy and negatively with helplessness, risk of cardiovascular disease, chronic physical diseases with age (Keyes, 2007). Flourishing individuals are more likely to function positively in different contexts.

## Resilience as a Mediator

Resilience is an important concept that attracts a great deal of attention of researchers across different fields including psychology, sociology, education and economy. Resilience refers to one’s capability to “bounce back” or “recovery” from negative life events, resist to illness and flexibility to adapt the environment in order to maintain his or her physical and psychological well-being (Ryff & Singer, 2003; Smith et al., 2008). Resilience is also characterised as a functional personality characteristic that promotes individual thriving after extremely traumatic events (Bonanno, 2004).

Resilience is a critical individual characteristic in promotion of well-being and associated with a wide range of health-related outcomes. Research into resilience has showed that resilience is negatively associated with anxiety, perceived stress, depression, negative affect, and physical symptoms and that positively associated with positive affect both in clinical and non-clinical samples (Smith et al., 2008). Smith et al. (2008) also found that resilience is positively associated with optimism, purpose in life, social support, active coping, and positive reframing and negatively associated with pessimism, alexithymia, negative interaction, behavioral disengagement, denial, and self-blame. Individuals with higher levels of resilience are more likely to experience less mental, social, and physical health problems. Some studies have explicitly explored the relationship between resilience and subjective and psychological well-being. For example, Sagone and De Caroli (2014) showed that resilience is positively related with autonomy, environmental mastery, positive relations with others, personal growth, purpose in life, and self-acceptance. Higher scores on resilience reflect greater psychological well-being. Shi et al. (2015) conducted a cross-sectional study of the mediating role of resilience in the relationship between stress and life satisfaction in Chinese medical students and found that resilience partially mediates the relationship between stress and life satisfaction. Higher scores on perceived stress are negatively associated with lower scores on resilience, resulting in lower level of life satisfaction.

Individuals with high levels of fear of happiness are confronted with various problems when they experience life challenges. On these occasions, the individuals’ levels of affect balance, satisfaction with life, and flourishing may be negatively affected. Previous studies have indicated the direct relationships between fear of happiness and well-being indices (e.g. Yildirim & Belen, 2018). However, due to the nature of the variables, the relationship between fear of happiness and well-being might be much more complex than a simple direct relationship. There might be other factors influencing the relationships between fear of happiness and well-being. Resilience might be a variable with the potential to explain the relationship between fear of happiness and well-being indices. Considering the negative effect of fear of happiness on individuals’ levels of well-being (Joshanloo, 2017; Yildirim et al., 2018), resilience might play an important role in protecting well-being against the negative effect of fear of happiness. To put it differently, resilience may function to preserve and promote positive psychological outcomes against negative impact of fear of happiness. Hence, examining the mediating role of resilience in the relationships between fear of happiness and indices of well-being would be fruitful to understand the mechanism underlying between the concepts. This would also assist to expand theoretical and empirical findings on fear of happiness, well-being, and resilience.

## Present Study

**Figure 1:**
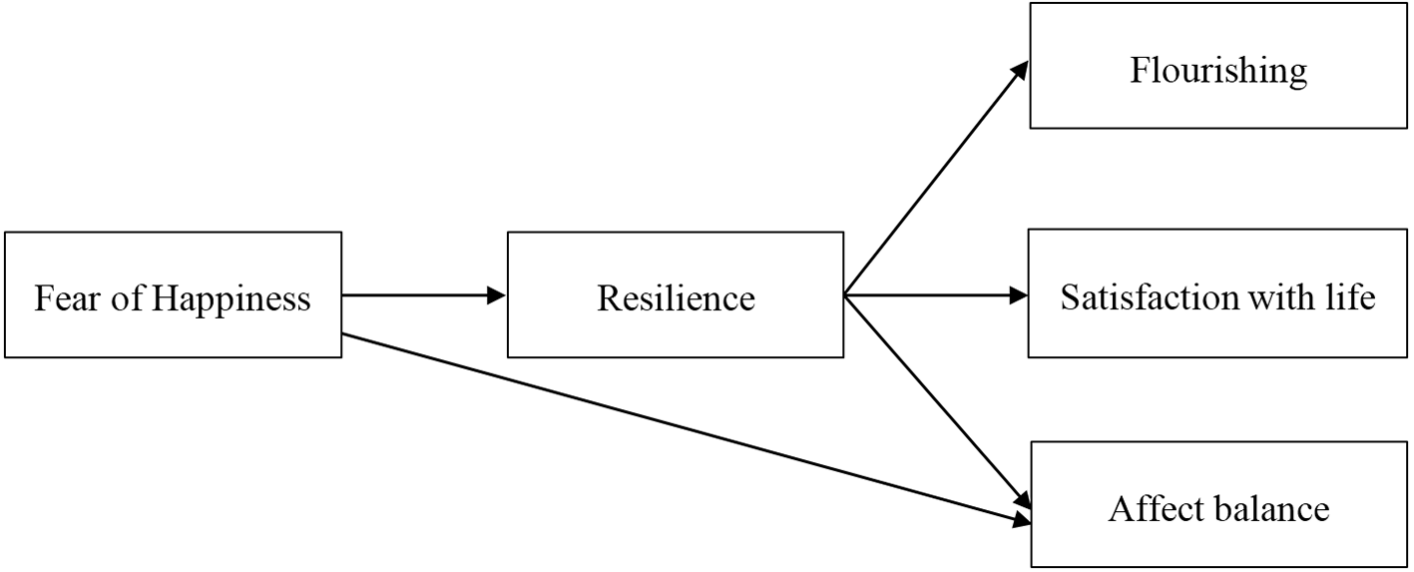
Hypothesised model used to analyse the impact of the resilience in the relationships between fear of happiness and affect balance, satisfaction with life, and flourishing.

Considering the role of resilience as preventive and protective factor in promotion of well-being, examining the mediating role of resilience on the relationship between fear of happiness and well-being can elucidate the mechanisms behind this association. The present study is important in terms of being the first study to investigate the impact of resilience in the relationship between fear of happiness and well-being indices. More specifically, the present study was set out to examine the impact of resilience on the relationships between fear of happiness and satisfaction with life, affect balance, and flourishing. Accordingly, we expected that fear of happiness would share a significant negative correlation with resilience, satisfaction with life, positive affect, affect balance, and flourishing and a significant positive correlation with negative affect. We also expected that resilience would share a significant positive correlation with satisfaction with life, positive affect, affect balance, and flourishing and a significant negative correlation with negative affect. Additionally, we hypothesised that fear of happiness would directly contribute to explain variation in flourishing, satisfaction with life, affect balance, and resilience. Furthermore, we expected that resilience would be directly important to account for variance in flourishing, satisfaction with life and affect balance. As for the mediation role of resilience in the relationship between fear of happiness and satisfaction with life, affect balance, and flourishing, we expected that resilience would fully mediate the relationships between fear of happiness and satisfaction with life, and flourishing, and partially mediate the relationship between fear of happiness and affect balance (see Figure 1).

## Method

### Participants

Participants comprised of 256 Turkish adults drawn from the general public. The participants ranged in age from 18 to 62 years old (*M* = 36.97, *SD* = 9.02). Table 1 shows the socio-demographic characteristics of the sample.

**Table 1:** Socio-Demographic Characteristics of the Sample

Variable	Frequency	Percent
Gender		
Males	174	68.0
Females	82	32.0
Marital status		
Married	201	78.5
Single	52	20.3
Widowed	3	1.2
Education level		
Secondary school	7	2.7
High school	28	10.9
College	9	3.5
University	128	50.0
Post-graduate	84	32.9
Economic level		
Very low	24	9.4
Low	46	18.0
Average	167	65.2
High	17	6.6
Very high	2	0.8

In summary, 68% of the samples were males, and 32% were females. 78.5% of the participants self-identified themselves as married, 20.3% as single, and 1.2% as widowed. The participants were typically highly educated. The most common education levels that participants completed at the time of taking part the survey were respectively university graduate (50%), post-graduate (32.9%), high-school graduate (10.9%), college graduate (3.5%), and secondary-school graduate (2.7%). As for their economic levels, the participants (65.2%) generally perceived their self-status as medium, 18% as low, 9.4% as very low, 6.6% as high, and 0.8% as very high. The participants did not receive any type of compensation for their involvement in the study.

### Measures

#### Fear of Happiness Scale

The Fear of Happiness Scale (FOH), developed by Joshanloo (2013), is a 5-item self-report scale that is designed to be unidimensional. The scale measures the general idea that having extreme positive emotions may lead to negative outcomes. The items are rated on a 7-point scale, ranging from 1 (strongly disagree) to 7 (strongly agree). Some of the items are “Having lots of joy and fun causes bad things to happen” and “Excessive joy has some bad consequences.” The scale score is the sum of items, with higher scores indicating higher level of fear of happiness. Reliability and validity studies of Turkish adaptation of the scale indicated good evidence of reliability and validity (Yildirim & Aziz, 2017). In this study, Cronbach’s alpha coefficient for the FOH was .86.

#### Satisfaction With Life Scale

The Satisfaction with Life Scale (SWLS), developed by Diener et al. (1985), is a 5-item self-report scale that is constructed to be unidimensional. The scale assesses individual’s global judgements of life satisfaction. The items are rated on a 7-point scale, ranging from 1 (strongly disagree) to 7 (strongly agree). Some of the items are “In most ways my life is close to my ideal” and “I am satisfied with my life.” The scale score is the sum of items, with higher scores indicating higher level of life satisfaction. Turkish adaptation of the scale showed satisfactory evidence of reliability and validity (Durak, Senol-Durak, & Gencoz, 2010). In the present study, Cronbach’s alpha coefficient for the SWBS was .81.

#### Scales of Positive and Negative Experience

The Scales of Positive and Negative Experience (SPANE), developed by Diener et al. (2010), is two-six items subscales measuring individual positive and negative emotional experiences and feelings over the previous four weeks. The items are rated on a 5-point scale, ranging from 1 (very rarely or never) to 5 (very often or always). Some items are “positive”, “happy”, “negative” and “sad”. The scale scores for positive affect and negative affect subscales are separately summed, with higher scores showing frequent experiences of positive feelings and frequent experiences of negative feelings, respectively. Affect balance score can be obtained by subtracting the negative score from the positive score, with higher scores showing frequent experiences of positive feelings and infrequent experiences of negative feelings. Turkish adaptation of the scale indicated good evidence of reliability and validity (Telef, 2015). In the present study, Cronbach’s alpha coefficient for the positive affect and negative affect were respectively .82 and .84.

#### Flourishing Scale

The Flourishing Scale (FS), developed by Diener et al. (2010), is an 8-item scale that is established to be unidimensional. The scale measures a wide range of social-psychological well-being from important domains of human functioning including relationships, life purpose, self-esteem and optimism. The items are rated on a 7-point scale, ranging from 1 (strongly disagree) to 7 (strongly agree). Some items are “My social relationships are supportive and rewarding” and “I lead a purposeful and meaningful life.” The scale score is the sum of items, with higher scores referring to higher level of satisfaction in significant areas of functioning. Turkish adaptation of the scale revealed satisfactory evidence of reliability and validity (Telef, 2010). In the present study, Cronbach’s alpha coefficient for the FH was .85.

#### Brief Resilience Scale

The Brief Resilience Scale (BRS), developed by Smith et al. (2008), is a 6-item self-report scale that is designed to be unidimensional. The scale measures the ability to bounce back or recover from adversities, setbacks, and failures. The items are rated on a 5-point scale, ranging from 1 (strongly disagree) to 5 (strongly agree). Some of the items are “I tend to bounce back quickly after hard times” and “I tend to take a long time to get over set-backs in my life.” Scale scores are the mean of items, with reverse coding of negatively worded items. Higher scores indicate higher ability of resilience to bounce back from stress. Turkish adaptation of the scale demonstrated satisfactory evidence of reliability and validity (Doğan, 2015). In this study, Cronbach’s alpha coefficient for the BRS was .86.

### Procedure

All participants completed online versions of the scales as part of questionnaire batteries being conducted for other studies. A secured online software was utilized for the data collection. The questionnaires were electronically advertised, after creating a link whereby participants had to click the link to access the study. Participants were assured regarding protection and disposal of personal data and confidentiality at any stage. They were also guaranteed about anonymity whereby they were assigned a number which was not directly associated with their personal information in our records. Furthermore, the participants were given freedom to withdraw from the study at any point in time without giving any reason. Those who agreed to collaborate were only allowed to continue and those who did not agree to collaborate were automatically discontinued completing the survey due to the way that the survey was designed. Participation was voluntary and consent to participate was obtained from each participants through the first page of the online survey. Presentation of the questionnaires was same for all participants.

## Results

### Preliminary Analysis

Prior to the main analysis, a preliminary analysis was conducted to determine whether the data was suitable for the analysis. Univariate outlier was computed using *Z*-score test statistic (Tabachnick & Fidell, 2001). No case was identified as a univariate outlier as all Z-scores were within the range of +3.29 and -3.29. Multivariate outliers were estimated using Mahalanobis D^2^ test and no case was determined as multivariate outlier. Tolerance and variance inflation factor through standard linear regression analysis were used to test for multicollinearity among the independent variables. No multicollinearity issue in the data set was detected as variance inflation factor was not greater than 10 and tolerance factor did not approach zero. Skewness and kurtosis statistics were used to examine normality assumption. According to Curran, West, and Finch (1996), if skewness and kurtosis values fall within the range of +1 and -1, the data is normally distributed. Table 2 shows means, standard deviations, skewness and kurtosis values with corresponding standard errors for each of the variables used in the study. As seen in Table 2, all variables fall within the range of +1 and -1, proving very good normal univariate distribution.

**Table 2:** Descriptive Statistics of the Study Variables (N = 256)

Variable	*M*	*SD*	Skewness		Kurtosis	
			Statistic	*SE*	Statistic	*SE*
Fear of happiness	13.88	8.09	0.67	0.15	-0.59	0.30
Resilience	19.59	4.54	-0.26	0.15	0.15	0.30
Satisfaction with life	21.56	6.50	-0.45	0.15	-0.62	0.30
SPANE-Positive	19.33	4.26	-0.19	0.15	-0.70	0.30
SPANE-Negative	17.19	3.99	0.46	0.15	-0.21	0.30
Affect balance	2.14	7.73	-0.36	0.15	-0.53	0.30
Flourishing	40.04	8.06	-0.86	0.15	0.47	0.30

### Correlation Analysis

Pearson product-moment correlation coefficients were estimated among all the study variables used in the present study. Table 3 presents the correlation coefficients between the study variables. As shown in Table 3, fear of happiness shared a significant negative correlation with resilience, positive affect, affect balance, satisfaction with life, and flourishing and a significant positive correlation with negative affect. Resilience shared significant positive correlations with satisfaction with life, positive affect, affect balance, and flourishing and a significant negative correlation with negative affect.

**Table 3:** Correlations Among the Study Variables (N = 256)

Variable	1	2	3	4	5	6	7
1. Fear of happiness	–						
2. Resilience	-.241**	–					
3. SWL	-.126*	.296**	–				
4. SPANE-Positive	-.281**	.527**	.428**	–			
5. SPANE-Negative	.251**	-.516**	-.442**	-.755**	–		
6. Affect balance	-.285**	.557**	.465**	.941**	-.932**	–	
7. Flourishing	-.141*	.432**	.396**	.525**	-.472**	.533**	–

*Note.* SWL = satisfaction with life; SPANE = scale of positive and negative experience.

*p < .05. **p < .01.

### Mediation Analysis

Separate regression analyses were performed to investigate the effect of fear of happiness on affect balance, satisfaction with life, and flourishing through resilience. For the mediation analysis, we adapted procedures recommended by Baron and Kenny (1986). In that procedure, establishment of four steps are required for the mediational hypothesis. In the first step, the independent variable should be significantly associated with the dependent variable. In the second step, the independent variable should be significantly associated with the proposed mediating variable. In the third step, the mediator variable should be significantly associated with the dependent variable. Finally, when the mediator is controlled for, a statistical significant reduction is required in the effect of independent variable on the dependent variable. If the effect is decreased to insignificant level, full mediation is present. If the effect is significantly decreased yet still remains significant, partial mediation is present.

To test the mediation analysis, three models were hypothesised. Model 1 suggested that resilience mediates the relationship between fear of happiness and satisfaction with life. Model 2 suggested that resilience mediates the relationship between fear of happiness and affect balance. Model 3 suggested that resilience mediates the relationship between fear of happiness and flourishing. We ran regression analyses to examine if resilience mediated the effect of fear of happiness on satisfaction with life, affect balance and flourishing. For each of the regression models, we considered satisfaction with life, affect balance, and flourishing as separate dependent variables and fear of happiness and resilience as independent variable and mediator, respectively.

The first regression analysis was conducted to examine whether fear of happiness significantly predicted dependent variables (e.g. satisfaction with life, affect balance, flourishing). The results indicated that fear of happiness had an unique effect on satisfaction with life, *R*^2^ = .02, B = -.10, 95% CI [-.20, -.00], β = -.13, *t* = -2.03, *p* = .043, affect balance, *R*^2^ = .08, B = -.27, 95% CI [-.39, -.16], β = -.29, *t* = -4.73, *p* < .001, and flourishing, *R*^2^ = .02, B = -.14, 95% CI [-.26, -.02], β = -.14, *t* = -2.27, *p* = .024. The second regression analysis was conducted to examine whether fear of happiness significantly predicted the mediator (e.g. resilience). The results indicated that fear of happiness was a significant predictor of resilience, *R*^2^ = .06, B = -.14, 95% CI [-.20, -.07], β = -.24, *t* = -3.96, *p* < .001. The third regression analysis was conducted to examine whether resilience significantly predicted dependent variables (e.g. satisfaction with life, affect balance, flourishing). The results indicated that resilience had an unique effect on satisfaction with life, *R*^2^ = .09, B = .42, 95% CI [.26, .59], β = .30, *t* = 4.92, *p* < .001, affect balance, *R*^2^ = .31, B = .95, 95% CI [.77, 1.12], β = .56, *t* = 10.70, *p* < .001, and flourishing, *R*^2^ = .19, B = .77, 95% CI [.57, .96], β = .43, *t* = 7.63, *p* < .001.

A series of separate hierarchical regression analysis were performed to examine the final step in mediational hypothesis. For each of the hierarchical regression analysis, fear of happiness and resilience were respectively treated as predictors in the Step 1 and Step 2, whereas satisfaction with life, affect balance, and flourishing were treated as dependent variables. Table 4 presents the impact of resilience in the relations between fear of happiness and satisfaction with life, affect balance, and flourishing.

**Table 4:** Mediating Role of Resilience in the Relationships Between Fear of Happiness and Satisfaction With Life, Affect Balance, and Flourishing

Predictor/outcome	B	*SE*	β	*t*	95% CI	*R*^2^	Δ*R*^2^
Satisfaction with life							
Step 1						**.02**	.02*
Fear of happiness	-0.10	0.05	-0.13	-2.03*	(-.20, -.00)		
Step 2						**.09**	.08***
Fear of happiness	-0.05	0.05	-0.06	-0.95	(-.15, .05)		
Resilience	0.40	0.09	0.28	4.57***	(.23, .58)		
Affect balance							
Step 1						**.08**	.08***
Fear of happiness	-0.27	0.06	-0.29	-4.73***	(-.39, -.16)		
Step 2						**.33**	.25***
Fear of happiness	-0.15	0.05	-0.16	-3.02**	(-.25, -.05)		
Resilience	0.88	0.09	0.52	9.82***	(.71, 1.06)		
Flourishing							
Step 1						**.02**	.02*
Fear of happiness	-0.14	0.06	-0.14	-2.27*	(-.26, -.02)		
Step 2						**.19**	.17***
Fear of happiness	-0.04	0.06	-0.04	-0.67	(-.15, .08)		
Resilience	0.75	0.10	0.42	7.24***	(.55, .95)		

*p < .05. **p < .01. ***p < .001.

The first hierarchical regression analysis revealed that in the Step 1, fear of happiness significantly predicted satisfaction with life, (*R*^2^ = .02, B = -.10, 95% CI [-.20, -.00], β = -.13, *t* = -2.03, *p* = .043). When resilience was included into the equation in the Step 2, the predictive effect of fear of happiness on satisfaction with life was reduced to insignificant, (B = -.05, 95% CI [-.15, .05], β = -.06, *t* = -.95, *p* = .345). Resilience, as a mediator, significantly predicted satisfaction with life even when fear of happiness was controlled for, (B = .40, 95% CI [.23, .58], β = .28, *t* = 4.57, *p* < .001). A Sobel test (Sobel, 1982) was performed to estimate whether the mediation effect was significant. The Sobel test showed a significant mediation effect of resilience, (*z* = -3.09, *p* < 0.01). This indicated that resilience has a full mediating effect on the relation between fear of happiness and satisfaction with life.

The second hierarchical regression analysis showed that in Step 1, fear of happiness significantly predicted affect balance, (*R*^2^ = .08, B = -.27, 95% CI [-.39, -.16], β = -.29, *t* = -4.73, *p* < .001). When resilience was added into the equation in the Step 2, the predictive effect of fear of happiness on affect balance was reduced yet significant, (B = -.15, 95% CI [-.25, -.05], β = -.16, *t* = -3.02, *p* = .003). Resilience, as a mediator, significantly predicted satisfaction with life even when fear of happiness was controlled for, (B = .88, 95% CI [.71, 1.06], β = .52, *t* = 9.82, *p* < .001). The Sobel test indicated that mediator effect of resilience in relation between fear of happiness and affect balance was statistically significant, (*z* = -3.72, *p* < 0.01). The results suggested that resilience partially mediated the relation between fear of happiness and affect balance. The results also suggested that fear of happiness contributed directly to account for variation in affect balance and indirectly through resilience.

The third hierarchical regression analysis showed that in Step 1, fear of happiness significantly predicted flourishing, (*R*^2^ = .02, B = -.14, 95% CI [-.26, -.02], β = -.14, *t* = -2.27, *p* = .024). When resilience was included into the equation in the Step 2, the predictive effect of fear of happiness on flourishing was reduced to insignificant, (B = -.04, 95% CI [-.15, .08], β = -.04, *t* = -.67, *p* = .503). Resilience, as a mediator, significantly predicted flourishing even when fear of happiness was controlled for, (B = .75, 95% CI [.55, .95], β = .42, *t* = 7.24, *p* < .001). The Sobel test showed a significant mediation effect of resilience, (*z =* -3.53, *p* < .01). This indicated that resilience has a full mediating effect on the relation between fear of happiness and flourishing.

## Discussion

The current study was the first study that examined the mediating role of resilience in the relationship between fear of happiness and satisfaction with life, affect balance, and flourishing. The results of the correlation analyses indicated that higher fear of happiness was significantly correlated with lower resilience, satisfaction with life, affect balance, and flourishing. These findings suggest that individuals with higher levels of fear of happiness are more likely to report poor ability to bounce back from stressful life events, subjective well-being, and psychological functioning. These findings are consistent with previous findings (e.g. Joshanloo, 2013; Yildirim & Aziz, 2017; Yildirim & Belen, 2018). For example, Yildirim and Belen (2018) found that higher fear of happiness significantly associated with lower subjective and psychological well-being. Indeed, the relationships between fear of happiness and resilience, and flourishing have not yet been examined. Considering flourishing as a psychosocial form of well-being (Diener et al., 2010; Keyes, 2002; Seligman, 2011) and its negative relation with maladaptive variables (e.g. depression), the emerging findings between fear of happiness, as a maladaptive variable, and flourishing is in the expected direction. Similarly, researchers (e.g. Joshanloo, 2017) proposed some dysfunctional happiness-related beliefs (e.g. externality of happiness, the idea that happiness is controlled by external resources) and found that resilience is negatively related to such beliefs and that mediates the relationships between such beliefs and well-being. Considering that fear of happiness is potentially a dysfunctional belief, the resulting findings are expected. The findings also suggested that higher resilience was associated with higher satisfaction with life, affect balance, and flourishing. These findings are in the line with relevant literature on resilience and subjective well-being, and flourishing (e.g. Keyes, 2002; Smith et al., 2008).

However, it is important to note that the effect sizes of the correlations in the present study are small based on Cohen’s (1992, 1988) criterion in which a value falls within .1 ≤ *r* < .3 reflects a small effect, a value within .3 ≤ *r* < .5 a medium effect, and a value of *r* > = .5 a large effect. Therefore, these results should be interpreted with caution.

Prior to testing the mediation models, we found that fear of happiness and resilience had significant direct effects on satisfaction with life, affect balance, and flourishing. The effects of fear of happiness and resilience on affect balance were lager in magnitude than the effect on satisfaction with life and flourishing. This variation may be related to the theory that affect balance rests on short-term life engagement in terms of experience of positive emotions and avoidance of pain and that satisfaction with life and psychological well-being or flourishing relies on long-term life engagement (Deci & Ryan, 2008; Keyes, Shmotkin, & Ryff, 2002; Ryan & Deci, 2001; Ryff & Keyes, 1995).

As to testing the mediation models, the results indicated that resilience fully mediated the relationships between fear of happiness and satisfaction with life, and flourishing. These findings suggest that as fear of happiness increases, satisfaction with life and flourishing decrease, and resilience directly affects this relationship. Additionally, the results showed that fear of happiness had not only direct effect on affect balance, but also indirect effect on it via resilience. Resilience was found to partially mediate the relationship between fear of happiness and affect balance. That is, individuals who scored high on fear of happiness had lower resilience, resulting in lower levels of affect balance, while individuals who scored low on fear of happiness had higher resilience, contributing to higher levels of affect balance. Higher fear of happiness also contributed to lower affect balance. These findings support the findings that fear of happiness may produce the tendency to hinder positive emotions, satisfaction with life and withdraw from social relationships that are important components to both subjective well-being and psychological well-being (Joshanloo, 2013; Joshanloo & Weijers, 2014), while produce the tendency to experience negative emotions such as anxiety, depression, and stress (Gilbert et al., 2012). Studies showed that having positive emotions is a typical characteristic of resilient individuals to “bounce back” from and find positive meaning in stressful situations quickly and effectively (Tugade & Fredrickson, 2004). Experiencing positive emotions may be a factor that individual use to foster their psychological health by buffering the negative impacts of fear of happiness.

Research suggested that subjective and psychological well-being or flourishing are conceptually different but related with one another and therefore it is necessary to explore both subjective and psychological well-being to fully understand well-being (Waterman, Schwartz, & Conti, 2008). In this study, therefore, we employed the influence of fear of happiness on both subjective well-being and flourishing via resilience. The emerging findings are important in terms of theoretical contribution to the extant literature and practical implications to health services. With regards to theoretical contribution, to the best of our knowledge, the present study provided the first evidence examining the impact of resilience in the relationships between fear of happiness and satisfaction with life, affect balance, and flourishing. This is meaningful to understand the mechanism underlying between fear of happiness and subjective well-being and optimal human functioning. The findings would also facilitate the theoretical improvement of the mechanism among fear of happiness, resilience and well-being. Concerning practical implication, because resilience was found to be a significant mediator in the relationship between fear of happiness and subjective well-being and flourishing, interventions aiming to enhance resilience may be useful in increasing positive affect, life satisfaction and flourishing and decreasing negative affect and fear of happiness.

There are several limitations of the current study that need to be acknowledged when the results are interpreted. The foremost limitation is that, the study was cross-sectional in nature limiting findings to draw a causal and definitive conclusion on the resulting relations among the variables. To achieve causal valid explanation of the results, assessment of the independent variable must be performed in advance of the mediator variable, and that assessment of the mediator variable must be performed in advance of the dependent variable (Jose, 2013). Hence, interpretation of the findings should be made cautiously. Studies employing prospective and longitudinal designs are needed to identify the causal links and provide evidence for the dynamic influence of fear of happiness on subjective well-being, and flourishing through resilience. Second, the study participants were predominantly male, married, university graduate, and having medium economic status. The findings may vary in populations with other demographic characteristics. Further research should investigate whether the obtained results can be replicated in other populations to enhance the generalizability of the findings. Third, a convenient sampling method was used to collect the data. Using a random sampling method where all participants have equal chance to participate the study would help to determine the effect of factors that may have been associated with the current sample (e.g. high rate of education level), that led to occurrence of the present findings.

In summary, the present findings indicated the importance of resilience in the relation between fear of happiness and satisfaction with life, affect balance, and flourishing. The results suggested that fear of happiness is associated with lower satisfaction with life, affect balance and flourishing as a result of lower resilience.
